# Transient Cortical Blindness: A Rare Sequelae Following Endovascular Embolization of a Basilar Tip Aneurysm

**DOI:** 10.7759/cureus.84348

**Published:** 2025-05-18

**Authors:** Vaisnavy Govindasamy, Divya Jacob

**Affiliations:** 1 Medicine, James Cook University Hospital, South Tees NHS Trust, Middlesbrough, GBR; 2 Ophthalmology, James Cook University Hospital, Middlesbrough, GBR

**Keywords:** contrast induced blindness, endovascular aneurysm repair, endovascular embolisation, posterior reversible encephalopathy syndrome (pres), skull bas and vascular neurosurgery, sudden vision loss, total cortical blindness

## Abstract

A 60-year-old woman with hypertension and a family history of aneurysms was found to have an incidental basilar tip aneurysm following a fall at Alicante airport. She had no history of polycystic kidney disease and was a non-smoker. The aneurysm measuring 1.2 cm was discovered following imaging after the head injury. The patient was counselled about treatment options, including endovascular embolization, which she opted for after understanding the cumulative risk of rupture. The procedure was performed via right common femoral artery (CFA) and left superficial femoral (SF) access under ultrasound guidance. Stents (2.25mm Leo Baby, Debene S.A., Buenos Aires, Argentina) were placed in both posterior cerebral arteries (PCAs), followed by subarachnoid (SA) coiling of the aneurysm. Post-procedure angiography confirmed no residual aneurysm filling, patent branches, and no thrombus formation.

Postoperatively, the patient developed bilateral vision loss and intermittent confusion. Neurological examination, including cranial nerve and ophthalmology assessment, was unremarkable except for anterograde amnesia. Arterial blood gas (ABG) analysis ruled out metabolic causes, and inflammatory markers and septic screening were unremarkable. Repeat MRI with diffusion-weighted imaging (DWI) sequences showed no acute infarction. Supportive management, including intravenous fluids, antithrombotic therapy, and symptomatic relief, was initiated. The patient’s vision improved from perception of light (PL) to hand motion (HM) bilaterally and fully recovered within 48 hours.

This case highlights transient cortical blindness as a possible complication following cerebral angiography. The primary differential was posterior reversible encephalopathy syndrome (PRES), but the absence of other symptoms such as headache, aphasia, facial numbness, seizures, ataxia, or visual hallucinations made transient cortical blindness the more likely etiology.

## Introduction

Basilar tip aneurysms pose significant risks due to their location and potential post-intervention complications. Endovascular embolization is the preferred treatment, yet post-procedural complications such as ischemia, thrombosis, and neurological deficits can occur [1]. Transient cortical blindness is an underrecognized but self-limited and reversible complication of cerebral angiography occurring in 0.3%-1.0% of cases, often within minutes to up to 12 hours after contrast medium injection [2-3].

Other potential causes of post-embolization vision loss include posterior reversible encephalopathy syndrome (PRES), contrast-induced neurotoxicity, and Anton syndrome. This report details a case of transient cortical blindness following embolization, emphasizing the need for awareness of this rare but reversible complication.

## Case presentation

**Figure 1:**
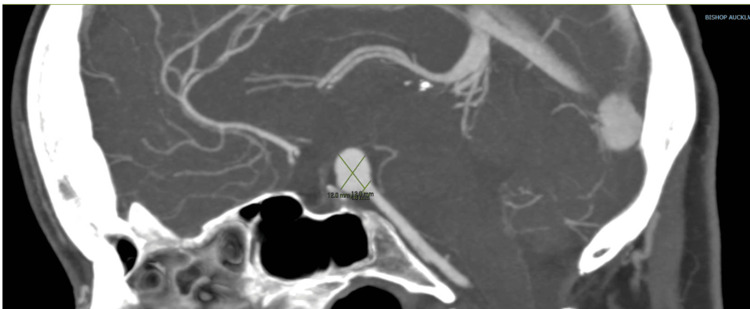
Represents CT brain of 12x12x4 mm basilar tip aneurysm on sagittal plane view

**Figure 2:**
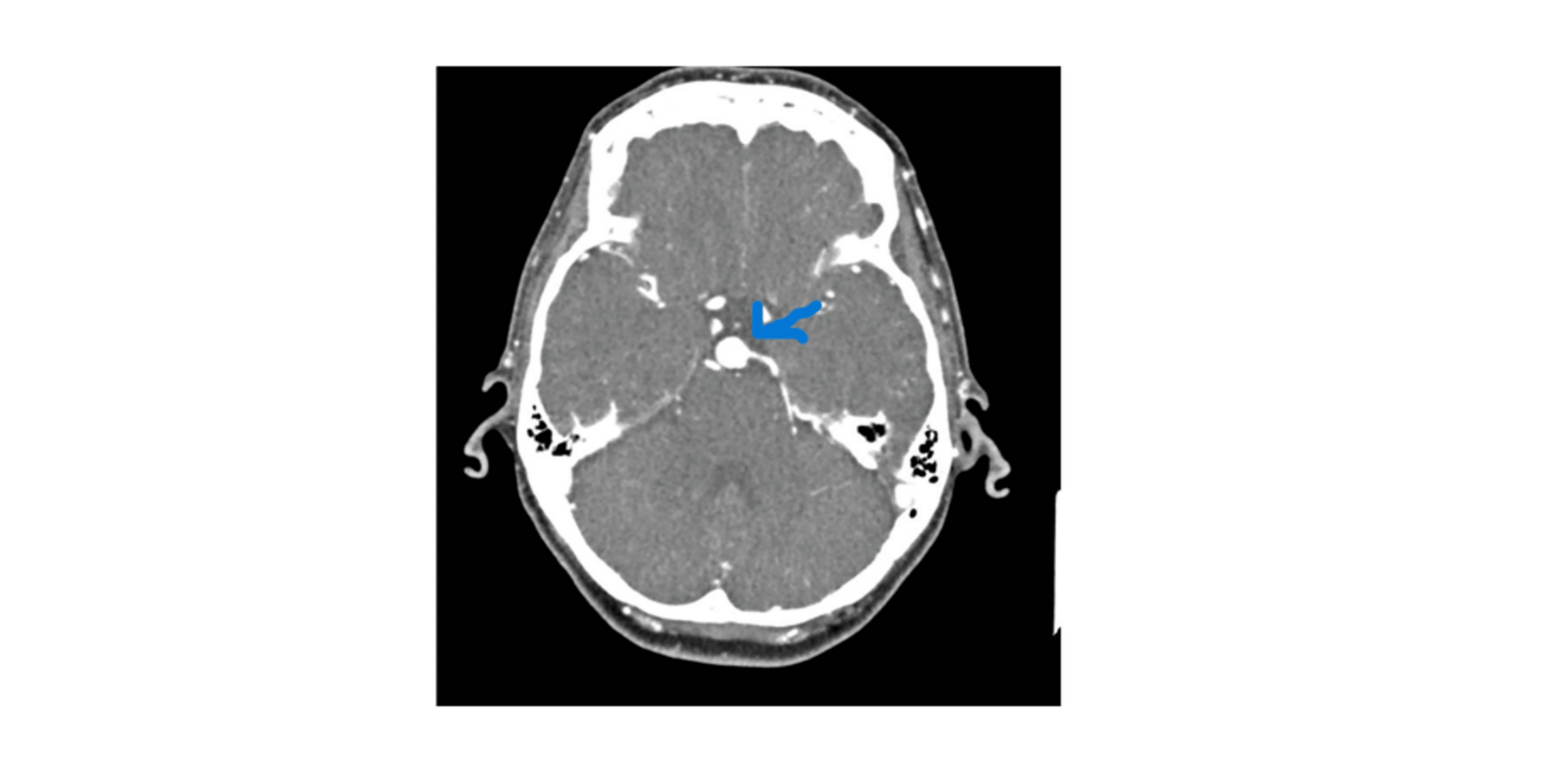
Represents CT brain of 12x12x4 mm basilar tip aneurysm on axial plane view

A 60-year-old woman suffered a traumatic brain injury, warranting neuro-imaging in January 2025. Her neuro-images, including CT angiography of the Circle of Willis, revealed an incidental basilar tip aneurysm approximately 1.2 cm, as shown in Figures 1 and 2. Her past medical history was significant for hypertension. She had no other risk factor for developing brain aneurysms apart from a positive family history (her father died of a ruptured brain aneurysm).

The patient was consulted on her treatment options, including conservative management or pre-emptive endovascular embolization with a detailed discussion regarding the low rupture risk of 0.5% per year for this aneurysm. However, according to the International Study of Unruptured Intracranial Aneurysms (ISUIA) study, the cumulative risk of rupture over time was considered to be 15%, leading her to opt for endovascular treatment [4].

Endovascular embolization was performed via the right common femoral artery and left superficial femoral access under ultrasound guidance. Stents (2.25 mm Leo Baby, Debene S.A., Buenos Aires, Argentina) were deployed in both posterior cerebral arteries (PCAs) of the basilar artery, followed by stent-assisted coiling of the aneurysm. Completion angiography confirmed complete aneurysm exclusion with patent vessels and no thrombus formation. The patient was monitored postoperatively, with immobilization of both lower limbs for four hours. She was initiated on prasugrel 5mg OD for three months and aspirin 75mg OD.

**Figure 3:**
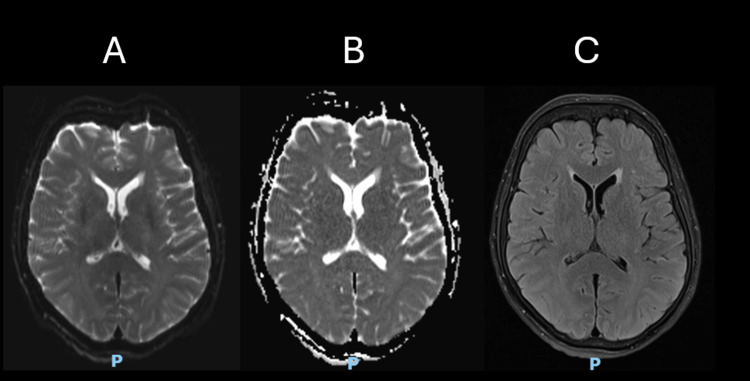
Represents MRI brain post-endovascular embolization axial plane view A) diffusion-weighted imaging, B) apparent diffusion coefficient, C) flair sequences

Within four hours following the procedure, the patient developed bilateral vision loss and intermittent confusion. Ophthalmology assessment revealed that visual acuity in both eyes was limited to hand movements. Examination showed normal eyelids, normal sclera, clear cornea, and a deep, quiet anterior chamber. The pupils were equal and reactive to light and accommodation. Both crystalline lenses were clear, with normal macula, fundus, and optic discs. Cranial nerve examination was otherwise normal, but anterograde amnesia was noted. ABG analysis ruled out metabolic causes, while inflammatory markers and infectious screening were negative. Post-procedural MRI with DWI sequences showed no evidence of stroke or infarction as illustrated in Figure 3.

Given the transient nature of symptoms, supportive imaging, and absence of PRES classical symptoms, a multidisciplinary team concluded that the findings were transient cortical blindness as opposed to PRES. Conservative management, including blood pressure control, IV fluids, and symptomatic relief, led to complete resolution of symptoms within 48 hours. The patient was subsequently discharged in stable condition.

## Discussion

Transient cortical blindness and its pathophysiology

Transient cortical blindness following cerebral angiography is a rare but well-documented phenomenon. While the exact pathophysiology remains speculative, two main mechanisms have been proposed. The first is contrast-induced neurotoxicity, which can lead to blood-brain barrier disruption and subsequent cortical dysfunction [5]. The second involves posterior reversible encephalopathy syndrome (PRES), characterised by perivascular edema that may impair visual processing within the occipital cortex [6]. In most cases, symptoms resolve spontaneously with cortical function typically normalising as the contrast agent is gradually cleared from the system.

Posterior reversible encephalopathy syndrome (PRES)

PRES is a neurological syndrome characterized by vasogenic edema in the posterior circulation, often triggered by hypertension, renal dysfunction, or iatrogenic factors such as contrast exposure during endovascular procedures [7]. PRES presents with headaches, altered mental status, seizures, and visual disturbances, including cortical blindness. Unlike transient cortical blindness, PRES can have more widespread neurological involvement and may require aggressive blood pressure control [8].

This differs from transient cortical blindness, which typically presents with isolated visual loss and lacks the systemic neurological symptoms such as seizures or altered mental status seen in PRES.

For instance, a recent study [9] reported PRES in a 56-year-old female following clipping of an unruptured intracranial aneurysm. Surgery proceeded without complications with stable intraoperative blood pressure and minimal blood loss. Postoperatively, the patient developed confusion and visual disturbances approximately six hours after extubation. MRI revealed hyperintense signals in the bilateral parieto-occipital regions, consistent with PRES. Blood pressure was elevated at this time, suggesting a hypertensive trigger. The patient's symptoms resolved with antihypertensive treatment and supportive care [9].

This differs from transient cortical blindness, where there is usually no confusion or hypertensive crisis, and neuroimaging does not typically show the extensive vasogenic edema seen in PRES.

Contrast-induced blindness

A rare complication that can occur after the administration of iodinated contrast agents during procedures such as cerebral angiography. It manifests as a sudden loss of vision due to the contrast medium disrupting the blood-brain barrier and affecting the occipital cortex. It is usually transient vision loss, often returning within hours to days. However, in some instances, the visual impairment may persist, causing permanent deficits that differ from transient cortical blindness [10].

Although similar in timing and presentation, contrast-induced blindness differs from transient cortical blindness as it results from direct neurotoxicity of contrast material rather than brief ischemia or metabolic disruption and may sometimes lead to irreversible visual deficits.

Anton syndrome

Anton syndrome is a rare condition characterized by cortical blindness with anosognosia, the patient's denial of their blindness. It occurs due to bilateral occipital lobe damage, often following strokes. Unlike the case described, Anton syndrome is typically persistent and associated with structural brain lesions on imaging [11].

In this case, the absence of other PRES symptoms, normal MRI findings, and rapid symptom resolution supported transient cortical blindness as the most likely aetiology. The patient's transient vision loss was likely due to contrast-induced neurotoxicity rather than ischemia or structural damage.

## Conclusions

This case underscores the importance of recognizing transient cortical blindness as a potential complication of neurovascular interventions. Awareness of this phenomenon allows for appropriate supportive management, reducing unnecessary investigations and interventions. Further research is needed to elucidate the underlying mechanisms and identify risk reduction strategies.
